# Risk Factors Associated with *Brucella* Seropositivity in Sheep and Goats in Duhok Province, Iraq

**DOI:** 10.3390/vetsci4040065

**Published:** 2017-12-07

**Authors:** Ali. G. Alhamada, Ihab Habib, Anne Barnes, Ian Robertson

**Affiliations:** 1 College of Veterinary Medicine, School of Veterinary and Life Sciences, Murdoch University, Perth 6150, Australia; gaddan76@gmail.com (A.G.A); a.barnes@murdoch.edu.au (A.B); i.robertson@murdoch.edu.au (I.R); 2 College of Veterinary Medicine, University of Mosul, Mosul 41002, Iraq; 3 High Institute of Public Health, Alexandria University, Alexandria 21516, Egypt; 4 China-Australia Joint Research and Training Center for Veterinary Epidemiology, Huazhong Agricultural University, Wuhan 430072, China

**Keywords:** brucellosis, small ruminants, abortion, risk factors, Iraq

## Abstract

Sera from 432 small ruminants (335 sheep and 97 goats) from 72 farms in Duhok Province, northern Iraq, were collected to investigate risk factors associated with brucellosis seropositivity. Serum samples were tested using the Rose Bengal test (RBT) and an indirect enzyme-linked immunosorbent assay (iELISA). Using parallel interpretation, RBT and iELISA results showed that 31.7% (95% confidence interval (CI): 26.1, 36.3) of sheep and 34.0% (95% CI: 24.7, 44.3) of goats had antibodies against *Brucella* in the study area. A random-effects multivariable logistic regression model indicated that a higher chance of being seropositive (odds ratio (OR) = 1.7; 95% 1.4; 2.2) was associated with an increase in the age of animals. The odds of *Brucella* seropositivity in flocks where sheep and goats grazed together was 2.0 times higher (95% CI: 1.08; 3.9) compared to flocks where sheep and goats grazed separately. The odds of *Brucella* seropositivity in small ruminants was 2.2 higher (95% CI: 1.2; 4.3) for animals originating from farms with a history of goat abortion in the preceding 12 months. In contrast, for every 1000 Iraqi Dinars (~0.85 US Dollar) spent by the farmers on control of *Brucella* in their flocks, the odds of *Brucella* seropositivity decreased significantly (OR = 0.9, *p*-value = 0.021). The final model also indicated significant differences in *Brucella* seropositivity between the different districts of Duhok Province. This study provides a contribution to the epidemiology of brucellosis in small ruminants in northern Iraq.

## 1. Introduction

Brucellosis is one of the most important types of zoonoses affecting both human and animal health. The disease is endemic in many nations throughout the Middle East, Mediterranean regions, Central Asia, and Latin America. *Brucella melitensis* (mainly infecting sheep and goats) is the most common cause of human brucellosis worldwide [1]. In humans, the disease manifests with acute febrile illness which, if not treated adequately, might develop complications that include chronic hepatomegaly, splenomegaly, and arthritis. In livestock, brucellosis mainly affects the reproductive organs and causes abortion, reduced fertility, and decreased milk production [2]. Hence, the disease could have serious negative socio-economic impacts on people, especially in low-income countries, due to loss of work or income as a consequence of illness and reduced profitability in the livestock sector [3].

In Iraq, the small ruminant (sheep and goats) sector is very important for sustaining the country’s food security. There are presently an estimated 7–8 million sheep and 1.5–2.0 million goats in Iraq, representing a valuable source of meat and milk production, and providing income and job security to people working across the agricultural sector [4]. An important challenge facing the small ruminant sector in Iraq is the challenging animal disease situation. Many endemic diseases are poorly managed and controlled as a consequence of the collapse of the veterinary infrastructure as a result of international economic sanctions and political and ethnic conflicts [5]. Among the many endemic animal diseases, *Brucella melitensis* continues to pose a threat to animal productivity and public health in Iraq. Jabary and Al-Samarraee [6] detected *Brucella* antibodies in 27.6% of whole blood samples (*n* = 311) from small ruminants in the Al-Sulaimanya governorate (north of Iraq). Many factors may play a role in the spread and survival of *Brucella* among animals, including variation in flock or herd size, animal density, and livestock contact between flocks [6,7]. The incidence of *Brucella melitensis* in humans in Iraq has been estimated to be between 52.3 cases per 100,000 person-years in a rural area to 268.8 cases per 100,000 person-years in a semi-rural area [8]. Such wide variations in reported brucellosis incidence is evident between different governorates in Iraq, highlighting the need to deepen our understanding of risk factors for disease transmission at the human-animal interface.

Northern provinces of Iraq share extensive, however loose, borders with neighboring Turkey and Syria. Brucellosis control in northern Iraq is very challenging, as it demands coordinated regional control efforts with neighboring countries [9]. Such coordination of control efforts is overshadowed by the political instability across the borders. For instance, Duhok province, at the very north of Iraq, has received a major influx of immigrants and refugees from neighboring Syria and from other parts of Iraq over the last two years [10]. This human migration also involved the movement of an estimated 100,000 sheep and goats. These livestock are often sold cheaply, grazed illegally, and not vaccinated regularly [10]. In such a setting, local livestock might become more vulnerable to unprecedented exposure, which might facilitate spread and persistence of many diseases. Therefore, objectives of the present study were to estimate the current seroprevalence of *Brucella* among sheep and goats in Duhok in the north of Iraq, and to identify risk factors associated with seropositivity.

## 2. Materials and Methods

### 2.1. Study Area and Population Included

Duhok province is located in the northern part of Iraq, and borders Syria and Turkey. The province is populated by approximately 1.2 million people, and contains about 1 million sheep and goats. Duhok is divided into seven districts, with each district being further subdivided into two sub-districts and a number of villages [11]. The study setting included six of the seven districts of Duhok; it was not possible to access one of the districts due to security concerns. The study was conducted between February and April 2016, preceding the governmentally subsidized *Brucella* vaccination campaign in the region, and thus omitting any biased seropositivity due to vaccination.

Duhok contains a large area of pastures, and sheep and goats are either grazed separately or together under a communal grazing system. Grazing of livestock is usually overseen by the farmers themselves or by shepherds employed by the farmers. A shepherd may be responsible for a large number of sheep and goats belonging to different owners. In this study, only flocks managed directly by farmers were included to ensure the accuracy of the questionnaire information. Flocks with a minimum of 100 animals (typical flock demographics in the study area) were eligible for inclusion in this study.

### 2.2. Sampling Strategy

There was no sampling frame available from the local veterinary office. Hence, we adopted a convenient two-stage sampling approach, based on a participatory approach with the help of local farmers’ knowledge and networks. We do realize that this might have affected the representativeness of the study population; however, such a limitation is inherently imposed by the immense logistical challenges of the field work area in Northern Iraq. All of the 12 sub-districts of the 6 surveyed districts in Duhok province were included in this study. Local community leaders in each district were approached at the central mosque, and were asked to voluntarily provide information about the farmers within the target sub-district who raised sheep and goats, and who met the inclusion criteria set for sampling (excluding shepherd-managed flocks, and excluding farms with <100 animals). From each sub-district, all farms meeting the study inclusion criteria for sampling (typically 2–3 farms), and whose owners agreed to participate in the study, were visited. A total of 432 individual blood samples (335 sheep and 97 goats) were collected. From each farm, six individual animals were randomly selected (random walk (every 10 steps, an animal was pointed at and then picked by farmer and taken to a holding pen)) for collection of blood samples. For mixed flocks, three sheep and three goats were sampled per farm. As mentioned before, sampling was achieved without probability proportional to size due to absence of farm registries in such remote study area. At the time of sampling, a questionnaire about the flock’s health and management was administered to the farmer.

A questionnaire was administered through a face-to-face interview to the selected farmers. The questionnaire was developed in English then translated into Kurdish and administered to the farmers by a native-speaking Kurd. Information was gathered about the management and husbandry practices adopted, flock make-up, and history of abortions in the relevant flocks (Tables 2 and 3).

### 2.3. Serological Analyses

Serum was extracted from whole blood by centrifugation at 3000 rpm for 10 min and stored at −20°C until testing. Each serum sample was screened for anti-*Brucella* antibodies using the Rose Bengal Test (RBT; VIRCELL, Granada, Spain) and commercial ELISA (NovaTec, Dietzenbach, Germany). The RBT was conducted according to the manufacturer’s protocol. Sera from sheep and goats were tested for anti-*Brucella* IgG antibodies using ELISA kits according to the manufacturer’s instructions and the recommended cut-off titer level. Testing was carried out in a nationally accredited commercial laboratory in Duhok. The serological status for given sera was given by a parallel test interpretation of the results of the two tests. Thus, serum was regarded as serologically positive when a positive result was recorded on one or both of the tests.

### 2.4. Statistical Analyses

The binary serological results (seronegative = 0/seropositive = 1) and variables on the characteristics of flock’s health and management, explained in the previous section, were recorded for all animals sampled for the study. The data were entered into an Excel spreadsheet and analyzed using STATA (Version 11.2, StataCorp, College Station, TX, USA). To account for the effect of the farms (categorical variable identifying the group structure) as a potential cluster variable, random-effects logistic regression models were used to assess the association between brucellosis seropositivity and predictor factors. The analysis was conducted in two steps. Firstly, the association between putative predictor factors (independent variables) and the dependent variable (*Brucella* seropositivity) was initially assessed using univariate logistic regression analysis to quantify the strength of association between the independent variables and *Brucella* seropositivity. The second stage in the analysis consisted of building a multivariable logistic regression model based on potential risk factors identified from the univariate analysis, with factors with a *p*-value ≤0.25 in the univariable analysis retained in the multivariable model. The most appropriate final model was selected using a backward stepwise selection approach. All pairwise interactions between the variables in the final model were examined for significance. Goodness of fit of the final model was assessed using the Hosmer–Lemeshow test. The associations between brucellosis seropositivity and putative risk factors were considered significant at a *p*-value ≤0.05, and were assessed using odds ratios (ORs) and 95% confidence intervals (CIs).

### 2.5. Ethical Approval

The study had been approved by the animal and human ethics committees of Murdoch University, with the permit number: R2805/15. All procedures were explained to farmers and informed verbal consent was obtained from all participants prior to sampling and administering the questionnaire.

## 3. Results

The seropositivity in goats (34%, 95% CI: 24.7, 44.3) was similar to that of sheep (31.7%, 95% CI: 26.1, 36.3) (*p* = 0.450). When a single test was used, only 71/137 (51.8%) and 102/137 (74.4%) were respectively classified as serologically positive by RBT and indirect enzyme-linked immunosorbent assay (iELISA) (Table 1). When the results of the two tests were interpreted in parallel, 137/432 (31.7%, 95% confidence interval (CI): 26.1, 36.3) sera were classified as serologically positive for antibodies against *Brucella*.

The results from the questionnaire and the corresponding serological results are summarized in Table 2 and Table 3 for categorical and continuous variables, respectively. Based on the univariate logistic regression analysis, the factors of age, districts, number of sheep per farm, number of goats per farm, flock grazing pattern, number of aborted goats on the farm during the preceding 12 months, farmer handling of aborted animals, sources of water on the farm, cleaning of water troughs, availability of agricultural workers on the farm, number of agricultural workers on the farm, and amount of money spent in flock on the control of *Brucella* in the preceding 12 months had a *p* value < 0.25, and were offered for the final random-effects multivariable logistic regression analysis (Table 4). The multivariable model indicated that none of the two-way interactions were statistically significant (*p* > 0.05). The Hosmer–Lemeshow test showed value of 3.065 (*p* = 0.930), which indicates a good fit of the model.

From the final model (Table 4), it could be suggested that the odds of brucellosis seropositivity were significantly higher with increasing age of animals (OR = 1.7; 95% CI: 1.4, 2.1). In addition, seropositivity among animals sampled from three districts was significantly lower (Amadiya (OR = 0.4; 95% CI: 0.1, 0.8), Duhok (OR = 0.3; 95% CI: 0.1, 0.7), and Shekhan (OR = OR = 0.4; 95% CI: 0.1, 0.8)) as compared to those from Aqarh (Table 4).The final multivariable logistic regression model indicated that the odds of seropositivity were 2 times (95% CI: 1.1, 3.9) higher for animals from flocks which grazed sheep and goats together, as compared to animals from flocks that did not graze sheep and goats together. Also, for goats on farms with abortions occurring in the 12 months preceding the survey, the odds for brucellosis seropositivity were 2.2 times (95% CI: 1.2, 4.3) higher compared to those from farms with no reported abortions in goats in the preceding 12 months (Table 4). For every 1000 Iraqi Dinars (~0.85 US Dollar) spent in the preceding 12 months to the survey on control of *Brucella* in small ruminant flocks in the study area, the odds of brucellosis seropositivity decreased significantly (OR = 0.93; 95% CI: 0.88, 0.98) (Table 4).

## 4. Discussion

In Iraq, brucellosis is considered as one of the most important endemic zoonotic diseases [12]. The main objective of this study was to investigate risk factors associated with brucellosis seropositivity among small ruminants reared in Duhok, northern Iraq. Result from this work provides the first pilot screening for *Brucella* in small ruminants, and also provides a better epidemiological insight that could be utilized for better management of such an important disease in animal production in the study setting in Iraq.

Seropositivity through the combined results of RBT and iELISA was used as the outcome variable for modeling risk factors in the study setting. The two tests used in this study are convenient and have been shown to be suitable for field screening. Nevertheless, none of the two tests are considered to be gold standard test and the approach of parallel interpretation has been widely recommended [13,14]. Despite being regarded as very sensitive, RBT and iELISA might still suffer from false-positive reactions due to presence of Gram-negative bacteria closely related to *Brucella* [14]. In this study, we combined both tests to achieve a parallel interpretation for the results. Our results revealed that about 25–50% of sera showing antibodies against brucellosis would have been classified as seronegative in a single testing approach either by RBT or iELISA. Several studies indicated that RBT is more suited for detecting the IgG1 and IgM typically produced during acute brucellosis infection. On the other hand, iELISA is more suited for detecting IgG which becomes dominant in chronic brucellosis cases [13,14]. Our results show that iELISA classified almost 25% more sera as seropositive, suggesting the predominance of a chronic infection context. Hence, a combination of tests could improve surveillance certainty and provide more reliable results for effective diagnosis and control of brucellosis.

In our study, the multivariable model analysis identified the age of animals as a risk factor associated with brucellosis seropositivity. In line with our finding, previous studies found that age has been regarded as one of the intrinsic factors to influence brucellosis seropositivity [15,16]. This could be attributed to the biological fact that clinical disease mainly affects the actively producing animals, as compared to young animals which have not reached reproductive age [17].

Next to animals’ age, the regression model indicated that brucellosis seropositivity was significantly higher among animals sampled from three of the districts (in Aqarh, Zakho, and Simele). Aqrah district, where 54.1% of the animals were seropositive, holds the main road for movement of animals and goods throughout the province. This might increase the likelihood of contact between local animals with others from different villages, and might cause an increase in the risk of transmission of *Brucella*. In addition, the three districts with significantly higher brucellosis seropositivity share the most borders with Syria, Turkey, and the Mosul province, where most of the uncontrolled movements of animals take place, provoked by war and political instability. Uncontrolled movement and smuggling of animals across borders could contribute to the spread and persistence of animal diseases in a regional context. In the neighboring country, Iran, Sharifi et al. [18] highlighted the importance of small ruminants with unknown history from neighboring countries, mostly from Syria, as a significant risk factor of seropositivity for *Brucella* in small ruminants.

In our study, brucellosis seropositivity was also found to be associated with sheep flock size, with mixed grazing between sheep and goats, and with history of goat abortion on a farm. The effect of flock size and mixed farming of multiple species on the risk of infections with contagious diseases has been well documented [19]. In our study area, the average size of sheep flocks was almost 4 times bigger than the average size of goat flocks. It could be hypothesized that the larger the flocks, the higher the chances for contact between individual animals, and in particular contact with an infected animal. Also, larger flocks will typically have greater chance of contracting other herds with subsequent transmission into the herd.

The use of communal pastures allows frequent contact between animals, and provides increased opportunity for environmental exposure to infectious materials, for instance arising from parturition. Previous studies have reported that contact between goats and sheep at the flock level was one of the most important risk factors for infection with *Brucella* [20,21]. Also, it has been documented that goats carry higher susceptibility to *Brucella* infection compared to sheep [22]. This is in line with our finding that abortion among goats on the farm in the preceding 12 months was a risk factor for brucellosis seropositivity in the current study setting. Abortion facilitates the release of an enormous number of microorganisms which can contaminate the environment and subsequently be ingested by at-risk healthy animals in the infected flock [23].

The regression model indicated that in the setting of Duhok, northern Iraq, for every 1000 Iraqi Dinar (~0.85 US Dollar) spent by the farmers on control of *Brucella* in their flocks, the odds of *Brucella* seropositivity decreased significantly (OR = 0.9, *p*-value = 0.021). This finding warrants for further investigation in order to elucidate further such a putative relationship. Farmers and their families would benefit from spending on brucellosis control at the farm level, for example by reducing mortality and morbidity costs and by opening up new trade opportunities. In addition, there is an obvious human health impact from expenditure on controlling brucellosis infection in the livestock sector. In Mongolia, research has found that if the costs of mass vaccination of livestock against brucellosis were allocated to all sectors in proportion to the benefits, the intervention would be profitable and cost-effective for the agricultural and health sectors [24]. Moreover, in Iraq, there have been several remarkable achievements attributed to past mass vaccination campaigns. The most important outcome was the apparent decrease in incidence of human brucellosis, which declined to almost 17 cases/100,000 people in the middle and south of Iraq in 2009 compared with 27.23 cases/100,000 in 2002 and 88.55 cases/100,000 in 1995 [25]. The positive results revealed a crucial need to continue vaccination procedures annually in order to reach the lowest possible incidence rate.

Despite the results of the study, some limitations were observed. First, there was the short period of field sampling, as this was concluded over 3 months. This was shorter than what we planned for initially, but we were forced to conclude the field research in a shorter time due to emerging security issues in the study area, which were beyond our control. Second, our inclusion criteria to sample from larger herds (at least 100 animals) could have impacted the seropositivity estimates. However, as this work is the first pilot study in the study area, we assume that targeting larger flocks was a reasonable starting point giving the immense challenges of field work in Northern Iraq and limitations in access to personnel support on the ground. Third, microbiological testing for confirming *Brucella* status was not possible to carry out under the study setting conditions, and certainly this could have helped to further confirm the status of the serologically-positive animals.

## 5. Conclusions

In this study we investigated risk factors for brucellosis seropositivity in small ruminates in Duhok province, northern Iraq, an area suffering from ongoing geopolitical and ethnic conflicts. This investigation revealed that the age of animals, districts from which animals are reared, sheep flock size, mixed grazing between sheep and goats, and history of goat abortion on farm in the preceding 12 months were independently associated with higher brucellosis seropositivity in small ruminants in the study area. The likelihood for seropositivity decreased with the increase of farmer’ expenditure on control of *Brucella* in sheep flocks. Given that the estimate of expenditure was directly provided by farmers, the possibility for a recall bias should not be ignored. We tried to validate the data provided by farmers regarding their estimate of expenditure; however, this was not possible to validate due to an absence of record-keeping on farms. Although the presence of antibodies does not necessary mean that sheep and goats are infected, these pilot results indicate the abundant presence of brucellosis in the study setting in northern Iraq. This study should be considered as a contribution to the epidemiology of brucellosis in small ruminants in Iraq.

## Figures and Tables

**Table 1:** Serological detection of antibodies to *Brucella* among 432 small ruminants (335 sheep and 97 goats) from Duhok Province (Iraq) using the Rose Bengal test (RBT) and indirect enzyme-linked immunosorbent assay (iELISA).

Sheep				Goats			
	ELISA (−)	ELISA (+)	TOTAL		ELISA (−)	ELISA (+)	TOTAL
RBT (−)	231	52	283	RBT (−)	64	14	78
RBT (+)	25	27	52	RBT (+)	10	9	19
	256	79	335		74	23	97

(+) positive; (−) negative.

**Table 2:** Categorical risk factors associated with brucellosis seropositivity among small ruminants from Duhok Province (Iraq) using parallel interpretation of the Rose Bengal test (RBT) and indirect enzyme-linked immunosorbent assay (iELISA).

Variables	Category	*n*	Seropositivity (%)
District	Aqarh	72	54.1
	Amadiya	72	22.2
	Duhok	72	20.8
	Simele	72	36.1
	Shekhan	72	26.4
	Zakho	72	38.9
Species	Sheep	335	31.0
	Goats	97	34.0
Sex	Male	45	28.1
	Female	387	32.0
Sheep vaccinated against *Brucella* in the preceding 12 months	Yes	211	31.1
	No	124	32.7
Goats vaccinated against *Brucella* in the preceding 12 months	Yes	53	30.5
	No	44	32.5
Flock grazing pattern	Sheep and goats graze together	252	33.3
	Sheep and goats graze separately	180	30.5
Flock grazing—Mixing of flocks from different farms	Yes	240	31.6
	No	192	31.7
Occurrence of abortion among animals on the farm in the preceding 12 months	Yes	228	35.5
	No	204	27.4
Farmer handling of aborted animals	Burning	6	50.0
	Given to dogs	348	33.9
	Thrown in public garbage	42	21.4
	Thrown into open water canals	36	19.4
Purchase (introduction) of new animals for the farm in the preceding 12 months	Yes	120	33.3
	No	312	31.1
Sources of water on the farm	River	66	33.3
	Well	132	31.8
	Spring	108	43.6
	Tap water	126	29.3
Water delivered to animals through:	Concrete troughs	36	30.5
	Metal troughs	396	31.8
	Wooden troughs	0	0
Cleaning of water troughs	Yes	420	31.0
	No	12	58.3
Sharing of water sources by sheep and goats with others from nearby farms	Yes	198	26.3
	No	234	36.3
Feed delivered to animals through:	Concrete troughs	36	22.2
	Metal troughs	360	32.7
	Wooden troughs	18	33.3
	Foraging	12	41.6
	Tires	6	0
Electricity on farm	Yes	204	29.1
	No	228	33.3
Availability of employed agricultural workers on the farm	Yes	33.3	27.1
	No	66.7	34.1

**Table 3:** Continuous risk factors associated with brucellosis seropositivity among small ruminants from Duhok Province (Iraq) using a parallel interpretation of the Rose Bengal test (RBT) and indirect enzyme-linked immunosorbent assay (iELISA).

Variable	Seropositive (Mean ± SD)	Seronegative (Mean ± SD)
Age (months)	4.6 ± 1.7	3.5 ± 1.4
Number of sheep on the farm	403 ± 550	533 ± 504
Number of goats on the farm	124 ± 176	94 ± 139
If mixed with other flocks while grazing—number of external flocks	2 ± 1.7	2 ± 1.6
Number of aborted sheep on farm during the preceding 12 months	20 ± 32.5	20 ± 33.2
Number of aborted goats on farm during the preceding 12 months	16 ± 20.0	12 ± 20.7
Number of agricultural workers on the farm	1.3 ± 3.7	2 ± 4.1
Amount of money spent on feed in the preceding 12 months (in 1000 Iraqi Dinars)	70 ± 9.3	60 ± 8.8
Amount of money spent on electricity in the preceding 12 months (in 1000 Iraqi Dinars)	10 ± 1.9	16 ± 3
Amount of money spent in flock on control of *Brucella* in the preceding 12 months (in 1000 Iraqi Dinars)	23.7 ± 36.3	39.4 ± 63.3

**Table 4:** Multivariable logistic regression analysis of risk factors associated with brucellosis seropositivity among small ruminants from Duhok Province (Iraq). CI: confidence interval; OR: odds ratio; S.E: standard error.

Variables		OR	95% CI	S.E.	*p*-Value
Age (month)		1.7	(1.4, 2.1)	0.196	<0.001
District	Aqarh	1.0	-	-	-
	Amadiya	0.4	(0.1, 0.8)	0.156	0.019
	Duhok	0.3	(0.1, 0.7)	0.145	0.012
	Simele	0.8	(0.4, 1.8)	0.335	0.670
	Shekhan	0.4	(0.1, 0.8)	0.156	0.019
	Zakho	1.4	(0.6, 3.2)	0.591	0.331
Number of sheep on the farm		1.0	(1.0, 1.1)	0.003	0.030
Flock grazing—Pattern	Sheep and goats graze separately	1.0	-	-	-
	Sheep and goats graze together	2.0	(1.1, 3.9)	0.684	0.028
Abortion among goats on farm in the preceding 12 months	No	1.0	-	-	-
	Yes	2.2	(1.2, 4.3)	0.742	0.012
Money spent (in 1000 Iraqi Dinars) on control of *Brucella* in the flock in the preceding 12 months		0.9	(0.8, 0.9)	0.002	0.021
